# Multiple Tolerances and Dye Decolorization Ability of a Novel Laccase Identified from *Staphylococcus Haemolyticus*

**DOI:** 10.4014/jmb.1910.10061

**Published:** 2020-01-23

**Authors:** Xingxing Li, Dongliang Liu, Zhaowei Wu, Dan Li, Yifei Cai, Yao Lu, Xin Zhao, Huping Xue

**Affiliations:** 1 College of Animal Science and Technology, Northwest A&F University, Yangling, Shaanxi 712100, P.R. China; 2 Department of Animal Science, McGill University, Quebec, Canada

**Keywords:** Laccase, staphylococci, dye decolorization, tolerance

## Abstract

Laccases are multicopper oxidases with important industrial value. In the study, a novel laccase gene (*mco*) in a *Staphylococcus haemolyticus* isolate is identified and heterologously expressed in *Escherichia coli*. Mco shares less than 40% of amino acid sequence identities with the other characterized laccases, exhibiting the maximal activity at pH 4.0 and 60°C with 2,2'-azino-bis (3-ethylbenzothiazoline-6-sulphonic acid) diammonium salt (ABTS) as a substrate. Additionally, the Mco is tolerant to a wide range of pH, heavy metal ions and many organic solvents, and it has a high decolorization capability toward textile dyes in the absence of redox mediators. The characteristics of the Mco make this laccase potentially useful for industrial applications such as textile finishing. Based on Blastn results, *mco* is found to be widely distributed in both the bacterial genome and bacterial plasmids. Its potential role in oxidative defense ability of staphylococci may contribute to the bacterial colonization and survival.

## Introduction

Laccases (benzenediol: oxygen oxidoreductase, E.C. 1.10.3.2), a group of copper-containing polyphenol oxidases, can oxidize a variety of phenolic and phenolate compounds by degrading them into smaller components and reduce molecular oxygen to water [1]. Due to their broad substrate specificity, laccases can be used for several industrial applications, such as dye decolorization in textile industries, detoxification of recalcitrant environmental pollutants, organic synthesis as a biocatalyst and bioremediation [1, 2]. The active site of laccases usually contains four copper ions [3], therefore the four copper binding regions L1, L2, L3, and L4 are considered as the standards for the identification of laccases [4].

In general, laccases are well-known enzymes widespread among fungi, especially ascomycetes, deuteromycetes and basidiomycetes. Fungal laccases usually show a higher redox potential than bacterial laccases [5, 6]. However, laccases derived from fungal origin unfortunately not only lose some of their activities in alkaline conditions but also are sensitive to high temperature as well as high salt concentration [7, 8]. Because of the lack of efficient heterologous expression hosts, the production of functional fungal laccases for industrial use is often difficult and needs a long time for cultivation [9]. Although site-directed mutagenesis and directed evolution are two major approaches to improving the performance of enzymes [10-12], expanding the pool to screen novel laccases is also of importance in the study. Recently, progress has been made in the investigation and isolation of prokaryotic laccases. Bacterial laccases may have potential to possess quite unique activities in physiology and biology at an extreme temperature and pH compared to the fungal laccases [13]. A laccase Lbh1 from *Bacillus halodurans* has been reported to exhibit chloride tolerance and show functional activity under the alkaline condition [14]. Another example involved the use of laccase HB27 derived from *Thermus thermophiles,* proving that an apparently high thermostable laccase (the highest ever reported in published literature) can be resistant at 85°C and incubated for 10 min [15]. In addition, bacterial laccases can be easily expressed in heterologous hosts like *Escherichia coli*, and the identification of functional prokaryotic laccases may provide a new alternative for industrial and agricultural applications.

Gram-positive bacteria staphylococci mainly reside in mucous membranes of humans and animals. A recent study found that *S. aureus* has a great ability to decolorize the azo and triphenylmethane dyes. Nevertheless, laccase gene was not identified or characterized in this case [16]. In the present study, a novel laccase gene *mco* was identified in a staphylococcal isolate *S. haemolyticus* NW19A. Mco was reconstructed and heterologously expressed in *E. coli*. The Mco enzymatic activity and tolerances to temperature, pH, heavy metal ions, enzymatic inhibitors and organic solvents were investigated by using the 2,2’-azino-bis(3-ethylbenzthiazolinesulfonic acid) (ABTS) as a substrate. It has a high decolorization capability toward textile dyes. The three-dimensional (3D) structure of Mco was constructed to reveal a possible reason for its tolerances. The research presented herein provides a novel laccase Mco with a specific activity, thermal and pH stability, a wide substrate range and strong dye-decolorizing ability, which are of importance for industrial and agriculture processes such as biodegradation and bioremediation.

## Materials and Methods

### Microbial Strains, Media and Vectors

*S. haemolyticus* NW19A, an isolate from mastitis milk samples of Holstein cows was routinely maintained on Tryptic Soy Agar (TSA) at 37°C. *Escherichia coli* DH5α were used for cloning procedures. Chaperone Competent Cell pG-KJE8/BL21 and plasmid pCold I were used for heterologous expression.

### Cloning of *mco*, Vector Construction and Bioinformatics Analysis

Genomic DNA of *S. haemolyticus* NW19A was purified by using an EasyPure Genomic DNA Kit (China). The laccase-like gene *mco* was amplified by PCR using the primers *mco*-F (5’-TCCGAATTCGAAAGTAAGAATGAC ATGAT-3’) and *mco*-R (5’-GACAAGCTTTTAGTTTGTTACTTTTATTT-3’). The underlined sequences represent the recognition sites of restriction enzymes EcoRӀ and HindIII, respectively. The amplified DNA fragment was purified using a Gel Extraction Kit (Transgen, China). The plasmid pCold I and purified PCR products were digested with EcoRӀ and HindIII. Then the *mco* gene was cloned into plasmid pCold I and expressed as a His-tagged form. For bioinformatics analyses, other laccase protein sequences from different microbial species were aligned with Mco using the ClustalX program. The conserved domains of Mco were predicted and analyzed using the conserved domain database [17]. The Homology Analysis was carried out with the neighbor-joining method (Mega software, Version 6.0).

### Expression and Purification of the Recombinant Laccase Mco

The recombinant vector was transformed into the Chaperone Competent Cell *E. coli* pG-KJE8/BL21. Transformants were subsequently inoculated into 200 ml LB supplemented with 100 μg/ml ampicillin and incubated at 37°C and 220 rpm until the turbidity reached 0.5. Then, the culture was chilled to 15°C and added with 0.5 mg/ml L-arabinose, 5 ng/mL tetracycline and 0.1 mM IPTG in order to induce the expression of Mco. The culture was continuously incubated at 15°C for overnight. The cells were harvested by centrifugation at 12,000 g for 2 min and then resuspended in binding buffer (20 mM sodium phosphate, 15 mM imidazole, 500 mM NaCl, pH=7.4). The cells were lysed by sonication and the supernatant containing soluble recombinant protein Mco was gathered by centrifugation at 18,000 *g* for 30 min at 4°C. Purification was performed on the HisTrap HP column system (GE Heathcare, USA). The crude fractions were dialyzed in TGE buffer at 4°C for 8 h to remove imidazole and were concentrated with Amicon ultrafiltration (membrane cutoff 10 kDa, USA). Purification products were analyzed by SDS-PAGE to check the purity achieved in the purification procedure, using a 5% stacking gel and a 12% resolving gel. Protein bands were visualized by staining with Coomassie Brilliant Blue R-250. Protein concentration was determined using the Bradford Protein Assay Kit ( China) with bovine serum albumin as the standard. The purified laccase Mco was stored at -80°C for further experiment.

### Enzyme Assay and Enzymatic Properties of Mco

The ABTS (Sigma, USA) was used as a substrate to confirm the laccase activity, whose absorbance coefficient was: ε_420nm_ = 36,000 M^-1^ cm^-1^. One unit of enzyme activity (U) is defined as the amount of enzyme which oxidizes 1 μmol of substrate per minute under the condition of 30°C and pH 4.5. All of these laccase activity assays were determined in a reaction mixture at 150 μl in a 100 mM sodium acetate buffer (pH 4.5) at 30°C with ABTS at 1 mM as the substrate [18]. For the Michaelis-Menten kinetics assay, gradient concentrations of ABTS ranging from 50 μM to 1,000 μM and Mco (5.21 mg/l) were employed and *K*_m_ and *k*_cat_ values were analyzed by the GraphPad Prism (Version 5.0) based on Lineweaver-Burk plots. To investigate the optimal pH and temperature tolerance of Mco, a gradient of temperature range (30~70°C) and a broad range of pH (2.0~12.0) were applied. The optimal pH value was determined in 100 mM sodium acetate buffers (pH 2.0~6.0), 100 mM sodium phosphate buffers (pH 6.0~8.0) and a 100 mM glycine—NaOH buffer (pH 9.0~12.0). For pH and thermal tolerance assays, Mco was pre-treated under certain conditions for 30 min and 1 h respectively, as described previously and the activity was routinely measured. The effects of metal ions, organic solvents and inhibitors on laccase activity were tested using 1 mM ABTS as the substrate and Mco (5.21 mg/l) at a temperature of 25°C and pH 4.5 in sodium acetate buffer. These inhibitors included Co^3+^ (C°l_3_), Cu^2+^ (CuSO_4_), Fe^2+^ (FeCl_2_), Mn^2+^ (MnCl_2_), Ni^2+^ (NiSO_4_), Zn^2+^ (ZnSO_4_), and Ba^2+^ (BaCl_2_) at a final concentration of 20 mM, organic solvents (acetone, DMSO, methanol, ethanol at a final volume fraction of 10% or 30%), sodium dodecyl sulfate including SDS (final concentrations: 5 mM and 2 mM), EDTA (final concentrations: 5 mM and 20 mM), and DTT (2 mM) [18-20]. The purified enzyme was pre-incubated with the metal ion or inhibitor for 15 min and the remaining activity was measured. All experiments were carried out in triplicate.

### Decolorization Ability of Mco

The dye-decolorizing ability of laccase was evaluated using four analytically pure dyes (Sigma), up to a 15 h period. The reaction mixture respectively contained 50 mg/l congo red, brilliant green, bromophenol blue or 20 mg/l crystal violet, acetate buffer (25 mM, pH 4.5) and purified enzyme (0.05 U) [6]. The decolorizing ability was measured at 30°C after every 3 h interval by measuring the absorbance at 625 nm for brilliant green, 490 nm for congo red, 592 nm for bromophenol blue, and 584 nm for crystal violet. The decolorization of dye, expressed as dye decolorization (%), was calculated by using the following formula: decolorization (%) = [(Ci -Ct)/Ci] × 100, where, Ci: initial concentration of the dye, Ct: dye concentration along the time [21]. All assays were performed in triplicate.

### Modeling of Three-Dimensional Structure of Mco

Swiss-Model (https://www.swissmodel.expasy.org/) was used to create the three-dimensional structure of Mco. The template structure is 3nsf (PDB code), which shares the highest sequence identity (37.87%) with Mco among the proteins in the Protein Data Bank (PDB) (http://www.rcsb.org/).

## Results and Discussion

### Heterologous Production and Activity of a Recombinant Laccase Mco

**Fig. 1:**
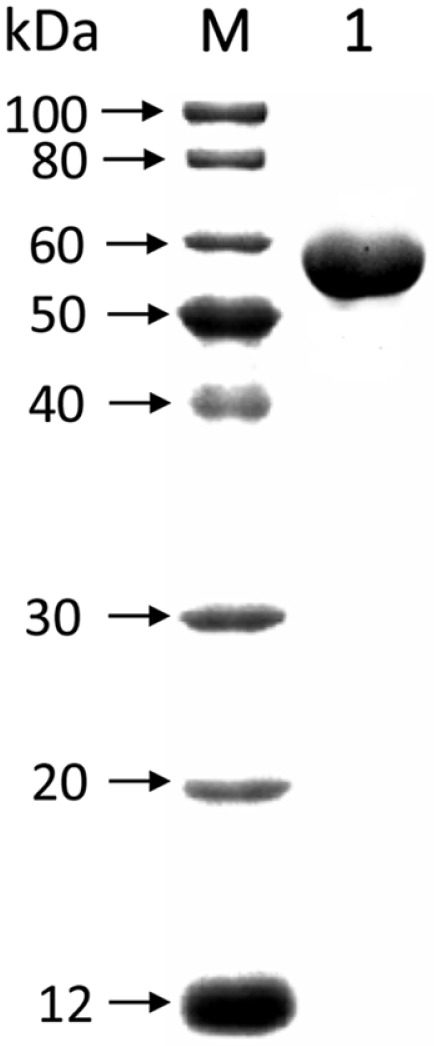
SDS-PAGE analysis of the recombinant laccase Mco from *S.haemolyticus* NW19A. Lane M: protein marker; lane 1: purified laccase Mco.

In order to identify the laccase activity of a multicopper oxidase from staphylococci, a 1,437bp ORF was amplified from the genome of *S. haemolyticus* NW19A and subsequently designated as *mco* (Fig. S1, GenBank Accession No. KM369884 (region: 61756-63192)) [22]. It encoded a protein with 478 amino acids and a molecular weight of 54.4 kDa (Fig. 1). Mco was expressed in *E. coli* and purified using nickel affinity chromatography. The purified Mco exhibited a specific activity of 34.0 U/mg when using ABTS as a substrate. According to the Lineweaver-Burk plots, *K*_m_ and *k*_cat_ values were 139.8 μM and 6.87 s^-1^, respectively, falling within the range compared with other bacterial laccases.

**Fig. 2:**
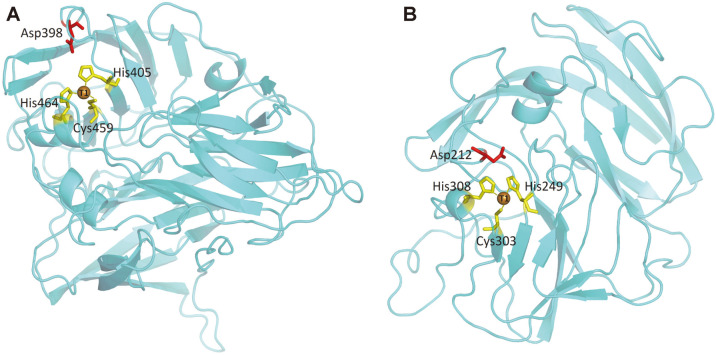
The predicted 3D structure of Mco (A) and Atm (B). The conserved histidine and systeine residues of the copper-binding motifs, as well as the aspartic acid residue Asp located in the vicinity of the substrate binding site cavity of laccases are shown.

The *K*_m_ for ABTS was 430 μM and 290 μM for two laccases from *Trametes versicolor*, with *V*_max_ values of 51.28 U/mg and 62.89 U/mg, respectively [23]. The *K*_m_ value of the laccase from basidiomycete *Fomitella fraxinea* for ABTS was 270 μM [24]. *K*_m_ value of an extracellular laccase from white-rot fungus *Marasmius scorodonius* was 27 μM for ABTS [25]. A laccase Lac1326 from a marine metagenomic library showed a *K*_m_ value of 210 μM for ABTS and a *V*_max_ value of 22.82 U/mg [26]. A Met-rich secondary structure was reported to occupy the T1 Cu site of many laccases and impair the ability to efficiently bind to the large substrate [10]. The Met-rich segment was absent in Mco, which may improve its substrate binding ability and catalytic activity (Fig. 2A).

### Thermal Stability and pH Stability of Mco

**Fig. 3:**
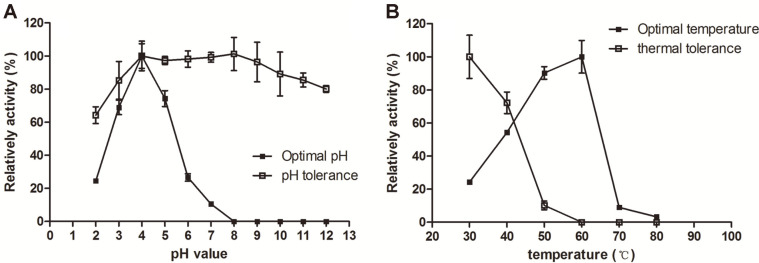
(A) The optimal pH and pH tolerance was evaluated at 30°C over a pH range of 2.0~12.0. (**B**) The optimal temperature and thermal tolerance of Mco were determined at various temperatures (30°~80°) and at pH 4.5. The activity measured at pH 4.5 and at 30°C was considered as 100%. Error bars represent the standard errors of the means.

The purified Mco exhibited its maximum activity at temperature 60°C and pH 4.0. The identified Mco tolerated a wider range of pH with more than 80% of enzymatic activity remaining between the ranges from pH 3.0 to pH 12.0 after 1 h treatment (Fig. 3A). Furthermore, the Mco retained approximately 72.2% of activity after incubation at 40°C for 30 min, but the activity was sharply reduced to 10.1% when the temperature was increased to 50°C. Almost no activity was observed at 60°C after 30 min of incubation (Fig. 3B). Enzymes with a high level of intrinsic thermal stability, which were prevailingly cloned from extremophiles, are desirable and suitable for industrial applications [15]. Staphylococci usually colonize in animals and their enzymes always work at 37°C, which might be a reason for their low thermal stability.

Generally, bacterial laccases show maximal catalytic rates in the neutral to alkaline pH range and fungal laccases show a preference for the acidic pH range [27]. Conserved acid amino acid residues Asp or Glu were located in the vicinity of the substrate binding site cavity of all known fungal laccases and were proposed to have a role in stabilizing the phenoxy radical formed during the catalytic reaction, but they were not present in any bacterial laccase identiﬁed so far [28]. In order to explore the possible reason why the optimal pH of Mco is acidic, the 3D structure of it was constructed, by utilizing a homology modeling approach via the Swiss-Model webserver. Not surprisingly, aspartic acid residue Asp398 was located in the vicinity of the substrate binding site cavity of Mco (Fig. 2A). Another bacterial originated laccase, Atm, identified by our lab, exhibited the maximal activity at pH 4.5 [29]. Amino acid residue Asp212 was also found in that site by modeling its 3D structure here (Fig. 2B). As far as we are concerned, it is the first time to reveal the possible reason for a bacterial laccase with acidic optimal pH.

### Tolerances of Mco to Heavy Metals, Enzyme Inhibitors and Organic Solvents

**Fig. 4:**
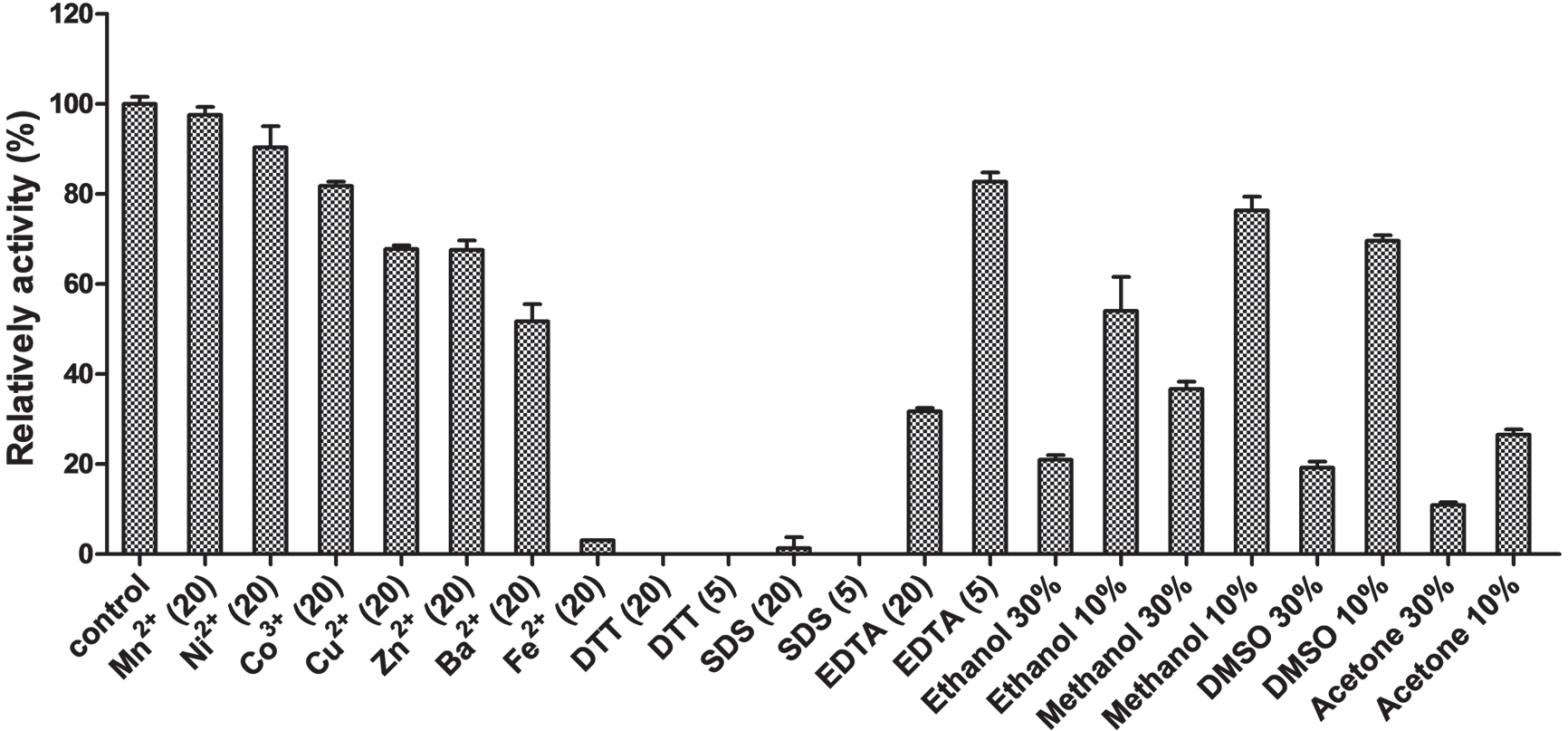
Effect of heavy metals (mM), organic solvents (%) and enzyme inhibitors (mM) on the enzymatic activity of Mco. Error bars represent the standard errors of the means.

Heavy metal ions, enzyme inhibitors and organic solvents are commonly used in many industrial processes and can influence the activity and stability of laccases. Consequently, the tolerances of Mco to heavy metals were determined. In the present study, the tolerances of Mco to heavy metals were assessed with the results shown in Fig. 4. No significant inhibition was observed for Mco with 20 mM Mn^2+^ used (97.5% remaining). The Mco activity was partially inhibited by Ni^2+^, Co^3+^, Cu^2+^, Zn^2+^, and Ba^2+^ (ranging from 51.7% to 90.3%) and completely inhibited by Fe^2+^ (3.1% remaining) at 20 mM. Furthermore, Mco exhibited excellent tolerance to EDTA, with 82.7% and 31.7% of the activity remaining at 5 mM and 20 mM of EDTA, respectively. This laccase maintained 54.0%, 76.3%, 69.6%, and 26.6% activities in the presence of 10% ethanol, methanol, DMSO and acetone, respectively. Moreover, the activities were not completely inhibited in all the tested solvents even at the concentrations of 30%. DTT and SDS completely inactivated the enzyme under the current test concentrations. Mco showed similar heavy metal tolerances to laccases from fungus *Fusarium solani* MAS2 and bacterial *Agrobacterium sp.* S5-1 for many metal ions, in addition, showed higher tolerances to Mn^2+^, Ni^2+^, and Co^3+^ than the laccase from MAS2 and higher tolerances to Ni^2+^ and Zn^2+^ than the laccase from S5-1 [18, 36]. The effect of metal ions on laccases depends on the binding site of metals and the size of the substrate binding cavity [15, 37, 38].

### Sequence Characteristics and Distribution of *mco*

**Fig. 5:**
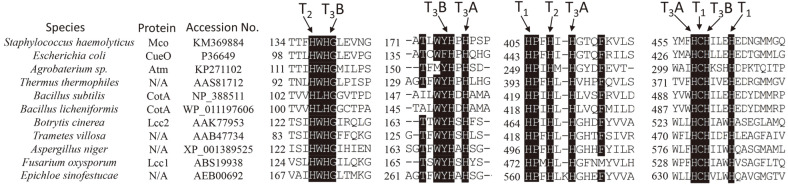
Multiple sequence alignment of the four copper-binding domains of Mco, several bacterial laccases, and fungal laccases. The four conserved copper-binding domains are indicated (boxed).

**Fig. 6:**
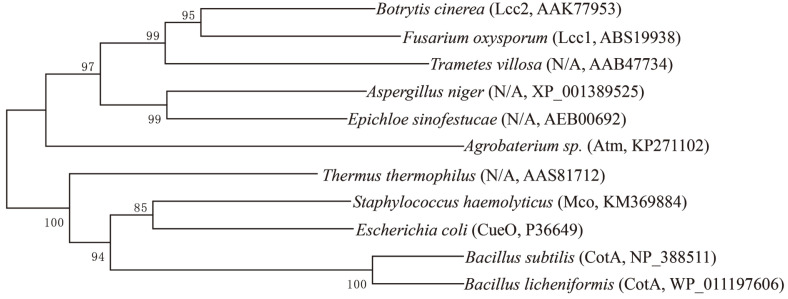
The phylogenetic analysis by a neighbor-joining method (Mega software, Version 6.0) utilizing the deduced amino acid sequences of Mco and other fungal and bacterial laccase protein sequences. Bootstrap values were obtained from 1000 repetitions and illustrated as percentages at the nodes. The evolutionary distance of 0.2 amino acid substitutions per position was represented at the scale bars. I and II represented laccases from fungi and bacteria, respectively.

Identifying the strictly conserved histidine residues of the copper-binding motifs is the main strategy to discover novel laccases. Four histidine-rich copper-binding domains were highly conserved in Mco in comparison with other bacterial and fungal laccases (Fig. 5) [30]. The phylogenetic relationship between Mco and other reported fungal as well as bacterial laccases was analyzed (Fig. 6). Mco shared the highest amino acid sequence identity of 35% with the characterized laccase CueO from *E. coli*. The overall similarity of the Mco to other bacterial laccases is low but higher than that to fungal laccases (Fig. 6).

Based on the Blastn results from the NCBI nr/nt database, *mco* was found to be widely distributed (Table S1). Among the top 49 matched sequences with the highest similarities to *mco*, 22 of them were located in bacterial genome (5 in *S. haemolyticus*, 7 in *S. epidermidis* and 10 in *S. aureus*) and 27 in bacterial plasmids (16 in *S. aureus* and 11 in *Listeria monocytogenes*). The presence of the *mco* gene in mobile genetic elements, such as staphylococcal cassette chromosome element and plasmids [22], is very important. It implies that the *mco* gene could be transferred horizontally among myriad kinds of staphylococcal isolates. The C-terminal segment of Mco in *S. haemolyticus* (amino acids 388 to 477) was absent in the Mco of *Listeria monocytogenes*. A C-terminus tail of 13-14 amino acids exhibits positive effects in the stability and laccase activity [10], and thus Mco in *Listeria monocytogenes* may perform low levels of stability and laccase activity. Low valence metal ions, such as Cu^+^ and Fe^2+^, generate toxic hydroxyl radicals in bacteria via the Fenton reaction [39]. The oxidation of them to high valence ions by laccase would protect bacteria against metal ion-promoted oxidative stress [39, 40]. The ability to mitigate endogenous and exogenous oxidative stress by obtaining a multitude of oxidative defense strategies accounts for staphylococci becoming a successful pathogen [41]. We propose that the laccase activity of Mco in staphylococci and its wide distribution in different staphylococcal species add a new oxidative defense strategy for staphylococci which should be paid attention to in future study.

### Ability of Mco on Decolorization

**Fig. 7:**
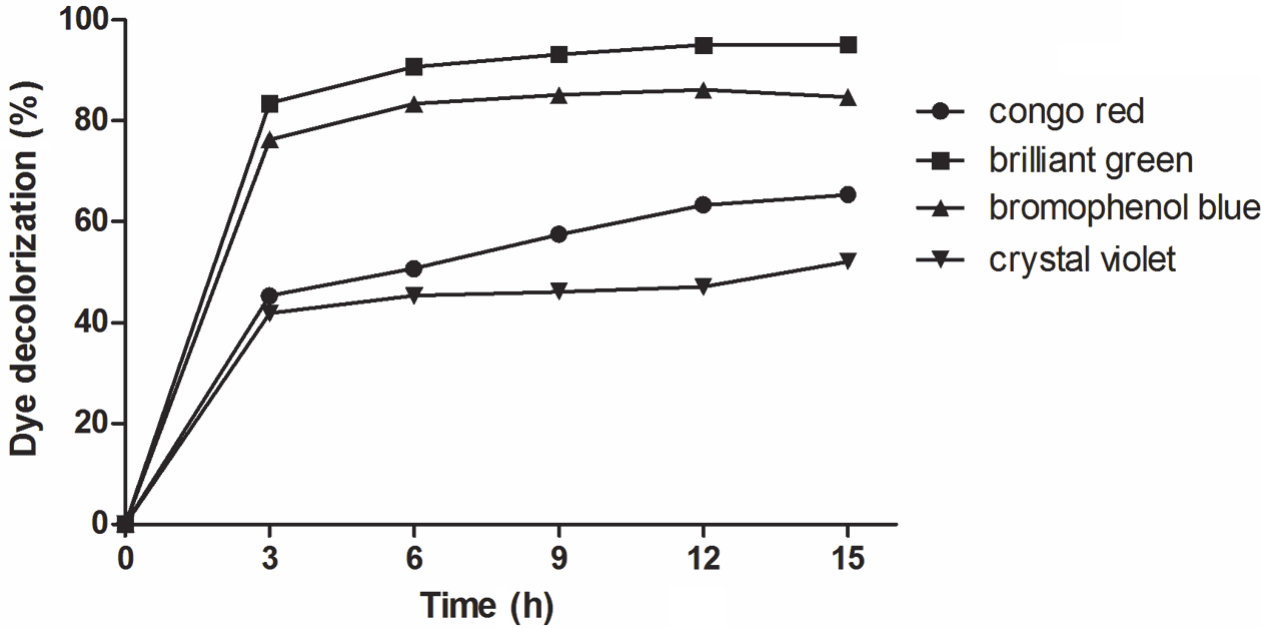
Decolorization of dyes by the purified Mco.

Decolorization of textile dyes is one of the most desirable applications for bacterial laccases [31]. Dyeing and textile industries account for a huge volume of highly colored dye effluents released into the environment. Triarylmethane and azo serve as the most commonly used dyes in the textile industry. Laccases can demethylate the triarylmethane or disassemble the azo bond of azo dye [1]. Therefore, the four representative dyes (three triarylmethane dyes as brilliant green, bromophenol blue and crystal violet and one azo dye congo red) were used to evaluate the decolorization ability of Mco (Fig. 7). Under the treatment of Mco at 3 h, more than 40% was decolorized for each dye. The decolorization efficiency of Mco to congo red, brilliant green, bromophenol blue and crystal violet after 15 h were 65.3%, 95.1%, 84.61%, and 52.1% in the absence of mediators, respectively.

In conclusion, the laccase has been isolated from a number of bacterial species including *Azospirillum lipoferum*, *Bacillus subtilis*, *Bordetella campestris*, *Caulobacter crescentus*, *Escherichia coli*, *Marinomonas mediterranea*, *Mycobacterium tuberculosum*, *Pseudomonas aeruginosa*, *Pseudomonas syringae*, *Stenotrophomonas maltophilia*, *Streptomyces griseus* and *Yersinia pestis*, but never from any staphylococci [2]. Hence, to the best of our knowledge, this Mco is the first identified laccase in staphylococci. The findings of our study showed that Mco exhibited high tolerances towards a wide range of pH, majorities of heavy metal ions and organic solvents. The high efficiency of dye decolorization by Mco was observed in the absence of mediators. Most mediators generate highly unstable radical intermediates toxic to the environment, and thus the addition of mediators in dye decolorization systems has no advantage in practical applications [32, 33]. The difference in the molecular structure of the dyes was proposed to be the reason for the variation in the decolorization efficiency [34]. These properties make this laccase Mco a suitable candidate for industrial applications such as textile finishing or wastewater treatment for energy-saving and low-cost purposes [32, 35]. Possible industrial applications of this novel enzyme should be explored in detail in a future study, including its ability to degrade a wider range of industrial dyes as well as substrate specificity. Its wide distribution and potential role in oxidative defense ability may contribute to staphylococci colonization and survival.

## Supplementary material

Supplementary data for this paper are available on-line only at http://jmb.or.kr.
