# Supplementary material for: Multiple Tolerances and Dye Decolorization Ability of a Novel Laccase Identified from Staphylococcus Haemolyticus
Source: J Microbiol Biotechnol. 2020 Jan 23;30(4):615–21. doi: 10.4014/jmb.1910.10061 (PMC9728370; doi:10.4014/jmb.1910.10061)
Supplement: Supplementary file 1 [file JMB-30-4-615-supple.pdf]

J. Microbiol. Biotechnol.
https://doi.org/10.4014/jmb.1910.10061

Table S1: Distribution of *mco* gene

Matched sequences	location	Identities	Species	Accession No.
NW19A	Genome	100%	*S. haemolyticus*	KM369884
SH32	Genome	100%	*S. haemolyticus*	KF006347
[illegible]1	Genome	100%	*S. haemolyticus*	JQ764731
[illegible]1435	Genome	99%	*S. haemolyticus*	AP006716
	Genome	99%	*S. haemolyticus*	CP013911
BPH0662	Genome	99%	*S. epidermidis*	LT571449
SEI	Genome	99%	*S. epidermidis*	CP009046
ATCC12228	Genome	99%	*S. epidermidis*	AE015929
949_S8	Genome	99%	*S. epidermidis*	CP010942
PM221	Genome	99%	*S. epidermidis*	HG813242
SR1	Genome	99%	*S. epidermidis*	AF270281
NTUH-7684	Genome	99%	*S. epidermidis*	LC085180
CA12	Genome	99%	*S. aureus*	CP007672
JCSC6945	Genome	99%	*S. aureus*	AB505630
JCSC6943	Genome	99%	*S. aureus*	AB505628
WA-MRSA-59	Genome	99%	*S. aureus*	KT316803
M121	Genome	99%	*S. aureus*	CP007670
RIVM3897	Genome	89%	*S. aureus*	CP013621
ATCC12600	Genome	89%	*S. aureus*	AY259130
71A_S11	Genome	88%	*S. aureus*	CP010940
FORC_001	Genome	88%	*S. aureus*	CP009554
MRSA252	Genome	88%	*S. aureus*	BX571856
pUR1902	Plasmid	89%	*S. aureus*	HF583291
pUR2941	Plasmid	89%	*S. aureus*	HF583290
pAF	Plasmid	89%	*S. aureus*	FN806789
SAP102A	Plasmid	89%	*S. aureus*	GQ900496
SAP075A	Plasmid	89%	*S. aureus*	GQ900486
SAP076A	Plasmid	89%	*S. aureus*	GQ900427
SAP077A	Plasmid	89%	*S. aureus*	GQ900428
SAP078A	Plasmid	89%	*S. aureus*	GQ900430
pFDA209P	Plasmid	88%	*S. aureus*	GQ900427
DSM20231	Plasmid	88%	*S. aureus*	CP011527
SAP067A	Plasmid	88%	*S. aureus*	GQ900483
SAP054A	Plasmid	88%	*S. aureus*	GQ900477
SAP019A	Plasmid	88%	*S. aureus*	GQ900385
pS1945	Plasmid	88%	*S. aureus*	CP009362
pUR2940	Plasmid	88%	*S. aureus*	HF583292
ATCC12600	Plasmid	88%	*S. aureus*	HQ663882
pLM-C-273	Plasmid	69%	*Listeria monocytogenes*	KX467250
pl2015TE24968	Plasmid	69%	*Listeria monocytogenes*	CP015985
pLmA144	Plasmid	69%	*Listeria monocytogenes*	KU513859
pLmN1546	Plasmid	69%	*Listeria monocytogenes*	CP013725
pLMR479a	Plasmid	69%	*Listeria monocytogenes*	HG813248
N1-011A	Plasmid	69%	*Listeria monocytogenes*	CP006611
R2-502	Plasmid	69%	*Listeria monocytogenes*	CP006595
pLM1-2bUG1	Plasmid	69%	*Listeria monocytogenes*	FR667692
pLM1-2cUG1	Plasmid	69%	*Listeria monocytogenes*	CP001603
pLM7UG1	Plasmid	69%	*Listeria monocytogenes*	FR667690
pLM5578	Plasmid	69%	*Listeria monocytogenes*	CP001603
